# A Glomus Tumor Presenting on the Ventromedial Aspect of the Little Finger Causing Bony Erosion: A Rare Case From India

**DOI:** 10.7759/cureus.54173

**Published:** 2024-02-14

**Authors:** Swaroop Solunke, Pratik T Gundecha, Archit Gupta, Rahul Salunkhe, Abhishek Nair

**Affiliations:** 1 Orthopaedics, Dr. D. Y. Patil Medical College, Hospital & Research Centre, Pune, IND; 2 Orthopaedic Surgery, Dr. D. Y. Patil Medical College, Hospital & Research Centre, Pune, IND

**Keywords:** glomus tumor, india, outcomes, bony erosion, glomangioma

## Abstract

Glomus tumors are rare neoplasms originating from the glomus body that predominantly manifest in the subungual region of the digits and are distinguished by severe pain and a heightened sensitivity to cold. Bony erosion associated with glomus tumors is a rare phenomenon. Here, we present a unique case of a glomus tumor situated on the ventromedial aspect of the little finger, leading to notable bony erosion. A 42-year-old female from India presented with a chief complaint of severe and localized pain in the ventromedial region of her right little finger, exacerbated by exposure to cold temperatures. Radiological investigations demonstrated focal bone erosion at the site of the tumor. Surgical excision of the lesion was performed. A fish-mouth incision was made on the ventromedial aspect of the little finger, which was extended to the tip of the finger. The nail bed was kept intact. The tumor was excised using small forceps. The patient experienced complete resolution of symptoms postoperatively and reported no recurrence during the follow-up period. This case report highlights the exceptional presentation of a glomus tumor causing bony erosion on the ventromedial aspect of the little finger, a manifestation rarely encountered in clinical practice. Furthermore, this case contributes to the limited body of literature on this combination of uncommon clinical entities, shedding light on its diagnosis and management.

## Introduction

Glomus tumors are rare benign vascular neoplasms frequently located within the hand, notably in the subungual region and pulp of the distal phalanx. Despite their infrequent occurrence, the precise etiology of these tumors remains largely elusive, prompting various hypotheses to elucidate their pathogenesis and the origin of associated pain. Characteristically, glomus tumors manifest as a bluish or pinkish-red discoloration of the nail plate and present with the classic triad of localized tenderness, intense pain, and heightened sensitivity to cold [1,2]. While often located in the subcutis and superficial soft tissues, glomus tumors can also manifest in various deep-seated and visceral sites throughout the body, such as the lungs, stomach, pancreas, liver, gastrointestinal tract, and genitourinary tract [3]. Complete surgical excision remains the definitive therapeutic approach, offering relief from symptoms and minimizing the likelihood of recurrence. Within the spectrum of glomus tumors, instances involving bony erosion represent a particularly uncommon and intriguing subset. While glomus tumors have been extensively documented [2,4], their capacity to induce structural changes in bone remains a seldom-explored phenomenon, with only a handful of cases reported [5,6]. The objective of this case report is to present a clinical encounter of a rare case of a glomus tumor situated on the ventromedial aspect of the little finger, culminating in significant bony erosion, which was excised using a unique surgical approach to salvage the nail bed.

## Case presentation

**Figure 1:**
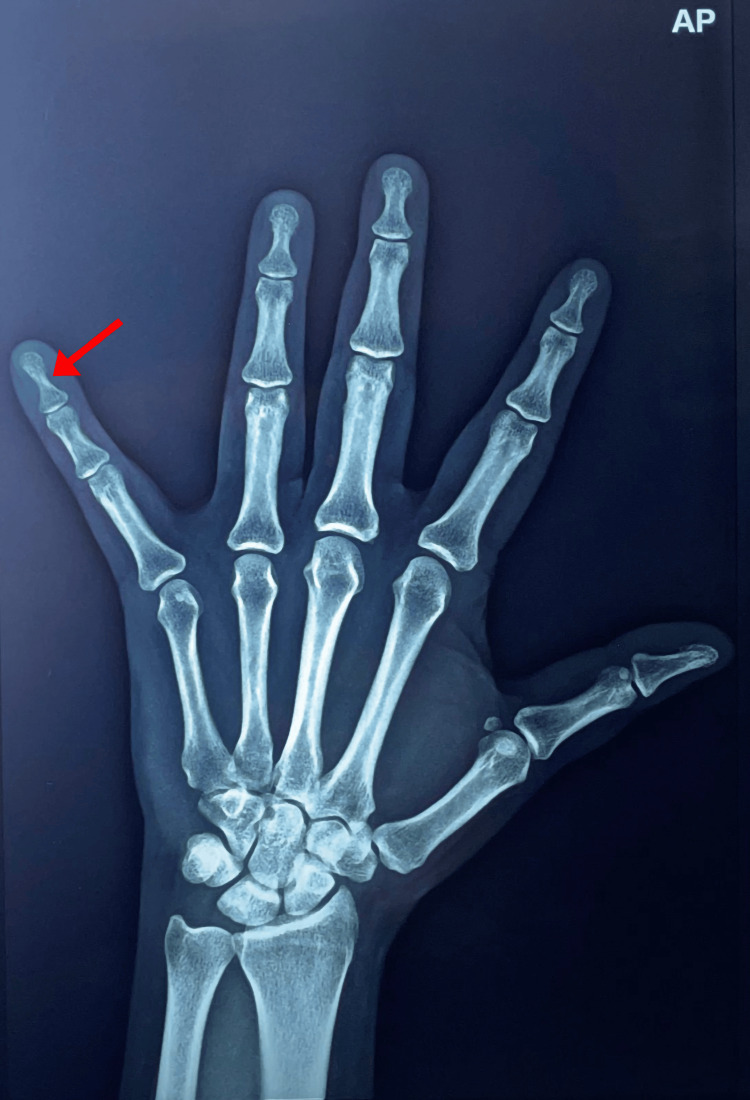
A plain radiograph of the little finger showing bony erosion over the ventromedial aspect of the distal phalanx of the little finger.

**Figure 2:**
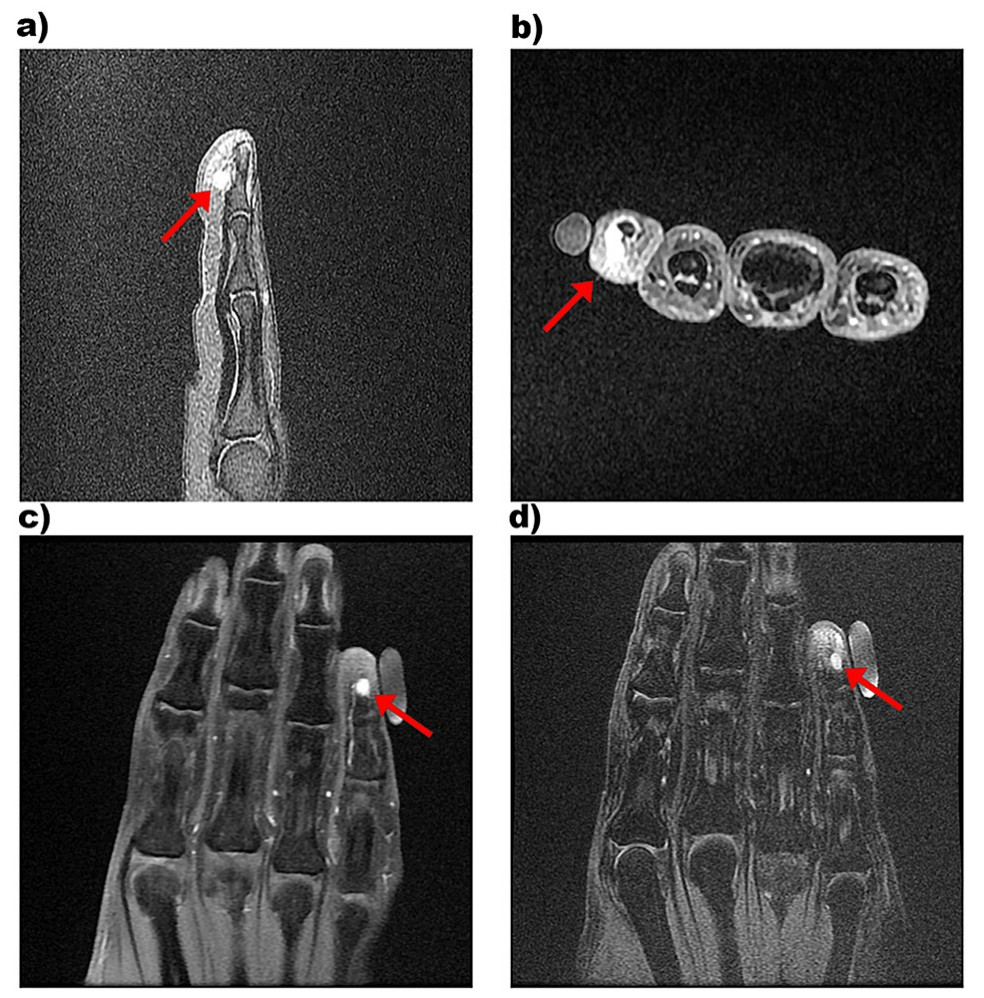
(a) Sagittal proton density (PD) fat-saturated image showing a hyperintense lesion on the ventral aspect of the distal phalanx of the fifth digit. (b) Axial post-contrast T1 fat-saturated image showing an intensely enhancing lesion. (c) Coronal post-contrast T1 fat-saturated image showing an intensely enhanced lesion. (d) Coronal PD fat-saturated image showing a hyperintense lesion on the ventral aspect of the distal phalanx of the fifth digit.

A 42-year-old female presented with complaints of pain over the ulnar aspect of the right little finger for four years. The pain was aggravated by exposure to cold environments and cold water. No history of trauma was reported. On examination, the cold sensitivity test was positive. There was no significant swelling or discoloration of the nail bed and surrounding soft tissue. A plain radiograph of the little finger showed bony erosion over the ventromedial aspect of the distal phalanx of the little finger (Figure 1). MRI was suggestive of a tiny, well-defined, oval-shaped T2 hyperintense and T1 hypointense lesion measuring about 6.1 x 4 x 2.8 mm along the ventromedial aspect of the distal phalanx of the little finger (Figure 2).

It was observed to cause smooth cortical scalloping of the distal phalanx. The lesion also showed intense post-contrast enhancement. Overall, the clinical and radiological features were suggestive of a glomus tumor.

**Figure 3:**
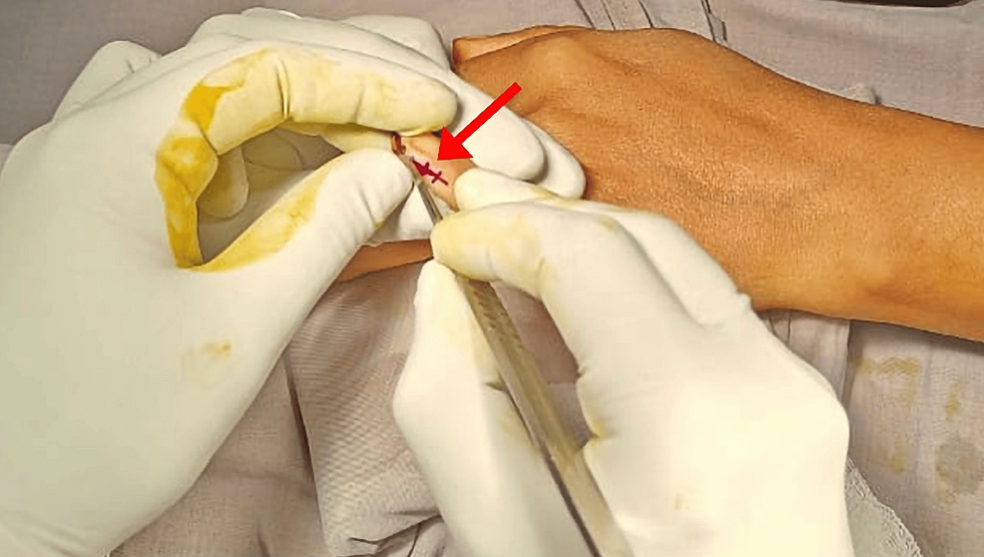
A fish-mouth incision was made on the ventromedial aspect of the little finger, which was extended to the tip of the finger.

**Figure 4:**
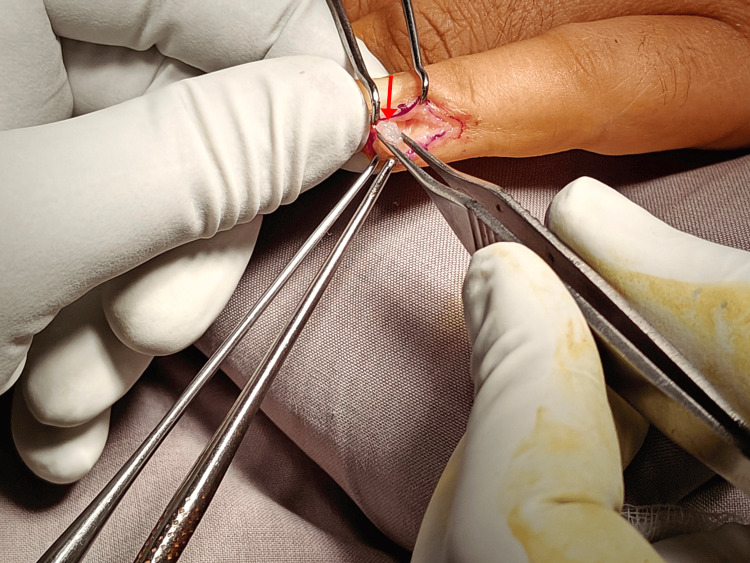
The nail bed was kept intact. The tumor was visualized following the superficial and deep dissection.

**Figure 5:**
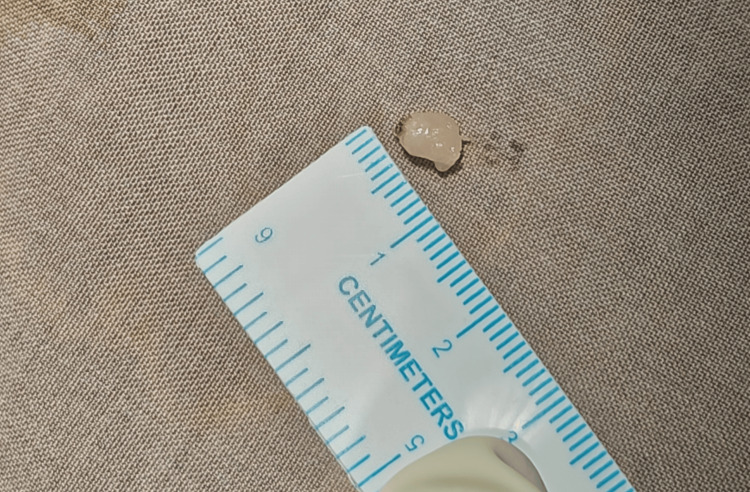
The size of the tumor was measured after the excision.

Considering the unusual site of the tumor presentation, i.e., the ventromedial aspect of the distal phalanx of the little finger, we adopted a unique surgical approach for managing this using small hand instruments. The surgical approaches were planned by keeping the vascular anatomy of the location in mind. A fish-mouth incision was made on the ventromedial aspect of the little finger, which was extended to the tip of the finger (Figures 3, 4). Using cautery and proper skin retractors, the tumor was excised using small forceps. Proper skin closure was done in layers. The size of the tumor was measured after the excision (Figure 5).

**Figure 6:**
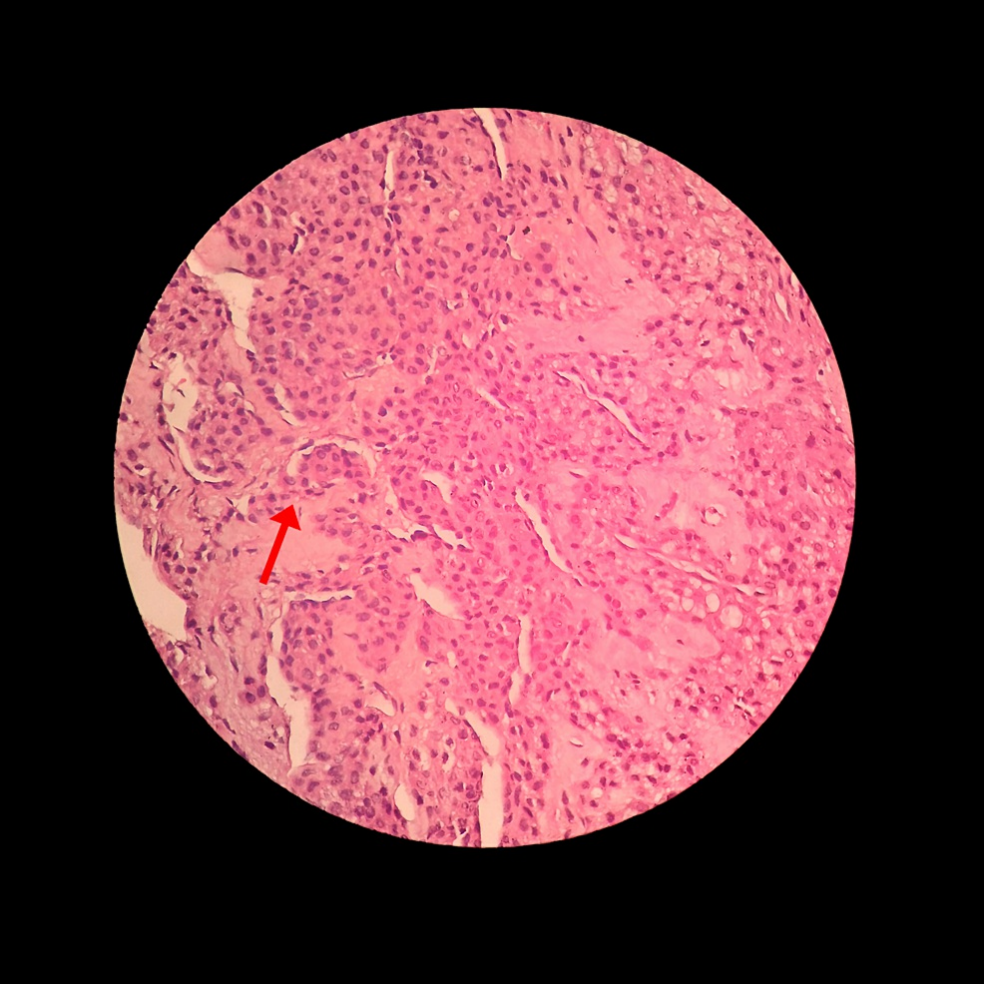
Rounded cells exhibiting vague boundaries and housing a round, sharply defined nucleus within a cytoplasm ranging from amphophilic to eosinophilic. The chromatin was uniform and unremarkable, with subtle nucleoli. Surrounding stroma was myxoid. Mitosis was not seen. No atypia/malignancy was seen.

The excised tumor was submitted for histopathological examination. Hematoxylin and eosin staining was done. The slide was seen under 500× magnification. Upon microscopic analysis, the sections displayed capillary-sized branching vessels, which were lined by endothelial cells and encircled by consistent glomus cells, constituting nests, sheets, and trabeculae. The cells were rounded, exhibiting vague boundaries, and housed a round, sharply defined nucleus within a cytoplasm that ranged from amphophilic to eosinophilic. The chromatin was uniform and unremarkable, with subtle nucleoli. Surrounding stroma was myxoid. Mitosis was not seen. No atypia/malignancy was seen (Figure 6).

These findings were consistent with the diagnosis of a benign glomus tumor. The patient had residual pain, which subsided slowly over the course of a month with tablet gabapentin (400 mg) + nortriptyline (10 mg) supplementation. The scar healed well on follow-up.

## Discussion

Glomus tumors, alternatively termed Barré-Masson syndrome, are rare, benign hamartomas arising from the glomus apparatus located within the subcutaneous tissues [7]. These tumors were first delineated by Wood in 1812, but their relationship with glomic origin was not confirmed until 1924 by Masson. Glomus bodies, pivotal for thermoregulation, are ubiquitously distributed throughout the human body. However, they are predominantly located in the distal digits, particularly under the nails; hence, the majority of glomus tumors are subungual [7]. Consequently, discovering a glomus tumor on the ventromedial aspect of a patient’s distal finger, as depicted in this instance, instead of beneath the nail bed, is uncommon [8]. Female preponderance is common in the subungual presentation of the glomus tumor [3]. Other factors such as age, trauma, or genetic inheritance are being considered in the etiology of the glomus tumor. Some theories propose that trauma may induce reactive hypertrophy in a weakened glomus body structure [2]. However, these risk factors were not evident in our case. Glomus tumors exhibit a variable evolution period, ranging from days to decades, and their rarity often results in delayed diagnoses and treatment initiation [9]. In the index case, the clinical symptom was present for four years. Studies have reported a mean duration ranging from 1.9 years to 10 years [10]. These tumors can be categorized as solitary or multiple, with the solitary type being more frequent and typically affecting middle-aged females, often localized to the distal phalanges of the fingers [9]. While 75% of glomus tumors are subungual [7], their occurrence over the ventromedial aspect of the distal phalanx, as seen in our patient, is rare. The cause of pain in glomus tumors remains unclear, with several hypotheses proposed. The capsules of the tumors may make them susceptible to pressure due to their structure. Moreover, the high concentration of mast cells present in glomus tumors can release substances such as heparin, 5-hydroxytryptamine, and histamine, which can sensitize receptors to pressure or stimuli from cold. Furthermore, the prevalence of multiple non-myelinated nerve fibers infiltrating the glomus tumors could be a contributing factor to pain sensations [2]. Diagnosing a glomus tumor primarily relies on patient history and clinical examination. Typically, glomus tumors manifest with a characteristic triad of symptoms: sensitivity to cold, increased pinprick sensitivity, and sporadic pain. These are often accompanied by unique subungual discoloration, hypoesthesia, atrophy, localized osteoporosis, and autonomic disturbances such as Horner syndrome [11]. Several clinical maneuvers assist in diagnosing these tumors. These include the Love pin test, which identifies localized pain in the lesion, with a positive result indicated by the patient retracting the finger due to pain; and the Hildreth test, a repetition of the Love pin test post-tourniquet application, in which the absence of pain is noted. The cold sensitivity test reveals an amplification of pain as temperature decreases, while transillumination and dermatoscopy of the nail plate offer further insights [2]. Dermoscopic evaluation of glomus tumors can be subtle, with nail plate dermoscopy revealing linear vascular structures in subungual tumors. However, these features can be subtle or absent, complicating the diagnosis [12].

Various imaging studies, including X-rays, CT, angiography, and ultrasonography, can offer accurate diagnostic insights. Radiographs can uncover cortical thinning or erosive alterations in neighboring bones, while MRI offers superior tissue contrast, illustrating a high-signal nidus encircled by a halo of lower signal intensity. MRI can play a significant role in the early diagnosis of the tumor with atypical signs and symptoms [2]. In the case presented, X-rays were sufficient to detect the mass in the distal finger, negating the necessity for additional radiological examinations. Although cost-effective and dynamic, ultrasonography can typically visualize lesions, commonly hypoechogenic, up to 3 mm in diameter [9]. Although essentially benign, glomus tumors can rarely coexist with sarcomas, leading to glomangiosarcoma [11].

Complete surgical excision remains the gold standard for treatment, with meticulous removal of all lesions to prevent recurrence, which can occur in 5-50% of cases, often due to incomplete excision [8]. The most common surgical approach is subungual excision by retracting the nail bed. However, the probability of complications in the nail is high following this procedure [13]. Modifications in this approach have been practiced for various levels and sites of the glomus tumor [2]. However, in our case, we removed the tumor without hampering the nail bed. Garg et al. reported a successful excision of a glomus tumor in their study of glomus tumors, where they also undertook a nail salvaging procedure, i.e., a “modified lateral subperiosteal approach” [4]. Following successful excision, patients typically experience rapid pain relief and restoration of normal finger appearance within three months [2], which was also exhibited by our patient.

## Conclusions

Glomus tumors, though rare, represent a clinical entity that demands vigilance and consideration in the evaluation of localized hand pain and discomfort. As demonstrated in this case, glomus tumors can occasionally deviate from their typical pattern and occur in unusual sites, such as the ventromedial aspect of the distal finger. The importance of meticulous and complete lesion removal cannot be overstated, as recurrence rates can vary significantly. Crucially, surgical excision remains the primary and most effective treatment modality, providing not only symptomatic relief but also minimizing the risk of recurrence. The modified nail-bed salvaging procedure done in the present case provides favorable outcomes in the unusually located glomus tumor associated with bony erosion.
